# Potent Dengue Virus Neutralization by a Therapeutic Antibody with Low Monovalent Affinity Requires Bivalent Engagement

**DOI:** 10.1371/journal.ppat.1004072

**Published:** 2014-04-17

**Authors:** Melissa A. Edeling, S. Kyle Austin, Bimmi Shrestha, Kimberly A. Dowd, Swati Mukherjee, Christopher A. Nelson, Syd Johnson, Manu N. Mabila, Elizabeth A. Christian, Joseph Rucker, Theodore C. Pierson, Michael S. Diamond, Daved H. Fremont

**Affiliations:** 1 Department of Pathology & Immunology, Washington University School of Medicine, St. Louis, Missouri, United States of America; 2 Viral Pathogenesis Section, Laboratory of Viral Diseases, National Institute of Allergy and Infectious Diseases, National Institutes of Health, Bethesda, Maryland, United States of America; 3 MacroGenics, Rockville, Maryland, United States of America; 4 Integral Molecular, Philadelphia, Pennsylvania, United States of America; 5 Department of Medicine, Washington University School of Medicine, St. Louis, Missouri, United States of America; 6 Department of Molecular Microbiology, Washington University School of Medicine, St. Louis, Missouri, United States of America; 7 Department of Biochemistry & Molecular Biophysics, Washington University School of Medicine, St. Louis, Missouri, United States of America; Institut Pasteur, France

## Abstract

We recently described our most potently neutralizing monoclonal antibody, E106, which protected against lethal Dengue virus type 1 (DENV-1) infection in mice. To further understand its functional properties, we determined the crystal structure of E106 Fab in complex with domain III (DIII) of DENV-1 envelope (E) protein to 2.45 Å resolution. Analysis of the complex revealed a small antibody-antigen interface with the epitope on DIII composed of nine residues along the lateral ridge and A-strand regions. Despite strong virus neutralizing activity of E106 IgG at picomolar concentrations, E106 Fab exhibited a ∼20,000-fold decrease in virus neutralization and bound isolated DIII, E, or viral particles with only a micromolar monovalent affinity. In comparison, E106 IgG bound DENV-1 virions with nanomolar avidity. The E106 epitope appears readily accessible on virions, as neutralization was largely temperature-independent. Collectively, our data suggest that E106 neutralizes DENV-1 infection through bivalent engagement of adjacent DIII subunits on a single virion. The isolation of anti-flavivirus antibodies that require bivalent binding to inhibit infection efficiently may be a rare event due to the unique icosahedral arrangement of envelope proteins on the virion surface.

## Author Summary

Dengue virus (DENV) is a globally important mosquito-transmitted human pathogen for which there is no approved vaccine or antiviral therapy. In recent years, the number and severity of DENV human infections have increased due to the expanded geographic range of the virus. Neutralizing antibodies are a key component of a protective natural and vaccine-induced immune response against human DENV infections. One recently described monoclonal antibody (E106) protects mice against infection of DENV-1 when administered before or several days after virus infection. Because of these results, we investigated the mechanism of action of E106 using a combination of structural and functional approaches. E106 engaged an epitope on domain III of the viral envelope protein that is a composite of two previously described epitopes. Unexpectedly, and in contrast to the intact IgG, Fab fragments of E106 were ineffective at neutralizing virus; this was explained by their weak micromolar affinity for virus particles. Our results suggest that neutralization by E106, our most potently inhibitory and protective anti-DENV MAb, requires bivalent binding of adjacent DIII subunits on a single virion. Immunization strategies with intact virions that skew the selection of neutralizing antibodies to those with bivalently binding properties could augment the potency of antiviral humoral responses against DENV and other flaviviruses.

## Introduction

Dengue virus (DENV) infection in humans causes symptoms ranging from a mild febrile illness to a severe and sometimes fatal disease. Over 3.6 billion people globally are at risk for DENV infection, with an estimated 390 million infections annually and no currently approved vaccine or antiviral therapy [1]. DENV belongs to the *Flaviviridae* family of medically important positive-stranded RNA viruses. Within the DENV serocomplex, there is significant diversity, including four serotypes (DENV-1, -2, -3, and 4) that differ at the amino acid level of the envelope (E) protein by ∼25 to 40 percent and multiple genotypes within a serotype that vary by up to ∼3 percent [2], [3].

A humoral response against DENV infection is believed to contribute to lifelong immunity against challenge by the homologous serotype. In comparison, protection against a heterologous DENV serotype infection is more transient (∼6 months to two years) [4], [5], allowing re-infection and disease to occur with a heterologous serotype in hyper-endemic areas of the world. Estimates suggest that greater than 90% of severe cases occur during secondary infection with a heterologous DENV serotype, possibly because sub-neutralizing amounts of cross-reactive antibody facilitate viral entry into myeloid cells expressing Fc-γ receptors, a phenomenon termed antibody-dependent enhancement of infection (ADE) [6]. Antibody-mediated protection against homologous DENV infection correlates with a neutralizing antibody response directed predominantly against the viral E protein [7]. The ectodomain of E is comprised of three domains: domain I (DI), a central nine-stranded β-barrel that connects domain II (DII), which contains the fusion peptide at its distal end, and an immunoglobulin-fold like domain III (DIII) [6]–[9]. Although neutralizing antibodies have been mapped to all three domains of the E protein, many potently inhibitory anti-DENV mouse MAbs map to DIII [10]–[17], specifically to the lateral ridge or A-strand epitopes, and block flavivirus infection at a post-attachment stage, likely by preventing E protein dimer-to-trimer transitions that are required for viral fusion [11], [15], [18].

We recently described a highly therapeutic MAb, DENV-1-E106 (hereafter termed E106), which neutralized infection of strains corresponding to all five DENV-1 genotypes and protected against lethal DENV-1 infection when administered as a single dose even four days after virus inoculation [13]. To understand the basis for the potency (plaque reduction neutralization titer (PRNT_50_) of 0.6 ng/ml) [13] and specificity of this MAb, we solved the crystal structure of E106 Fab in complex with DENV-1 E DIII. Our analysis revealed a small antibody-antigen interface with contact residues corresponding to two previously characterized DIII epitopes. Remarkably, a ∼20,000-fold disparity in neutralization by intact IgG and Fab correlated with distinct abilities to bind intact virions. Our results are consistent with a model in which our most potently inhibitory and therapeutic DENV-1 MAb requires bivalent binding through dual and simultaneous engagement of two antigen binding sites on a single virion to neutralize infection. E106 is therefore one of only a few unique antibodies described to date where effective neutralization requires a bivalent binding mechanism, and is the first such characterized MAb directed against flaviviruses.

## Results

### The crystal structure of E106 in complex with DIII reveals a small footprint

**Figure 1:**
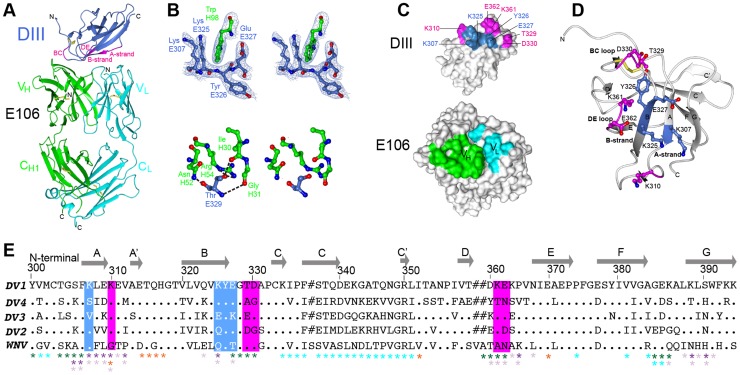
Crystal structure of E106 Fab in complex with DENV-1 E DIII. (**A**) The E106 Fab epitope on DENV-1 E DIII is comprised of residues in the A-strand (K307 and K310), the end of the B-strand (K325 and Y326), and the connecting BC (E327, T329, and D330) and DE (K361 and E362) loops. The immunoglobulin-like DIII is shown in blue (epitope regions in magenta), with the E106 heavy chain in green and light chain in cyan. (**B**) Heavy chain residue W98 binds in a deep pocket contributed by the aliphatic groups of side chain DENV-1 E DIII residues K307, K325, and E327 and main chain of Y326, in stereo (top panel). The electron density map is contoured at 1.1σ. Ball and stick representation of the molecular interactions involving T329 (the residue that escapes neutralization [13] in stereo (bottom panel). (**C**) Surface representation of DENV-1 E DIII (top) and E106 Fab (bottom) highlighting residues making direct contacts in the complex (see Table S1). DENV-1 E DIII residues previously identified by yeast surface display are displayed in magenta [13]. (**D**) The structural epitope on DENV-1 DIII is shown in ribbon representation. (**E**) Sequence of DIII of DENV-1 aligned with that of DENV-2, -3, -4 and WNV highlighting the E106 structural epitope, which is conserved in DENV-1 genotypes but not DENV serotypes or WNV. Identical residues are represented by a dot, deleted residues by a hash. For comparison, The DIII structural epitopes of WNV E16, DENV-2 1A1D-2, DENV cross-reactive 4E11 and 2H12 MAbs and DENV-1-E111 contact residues are labeled with green, purple, light purple, orange and cyan asterisks respectively [10], [17], [20], [21], [33].

E106 is a sub-complex specific therapeutic MAb that binds to DENV-1 and DENV-4 infected cells and neutralizes infection of all five DENV-1 genotypes efficiently (EC50 ranging from 1 to 50 ng/ml), without neutralizing DENV-2, 3 and 4 serotypes or WNV [13]. The 2.45 Å crystal structure of E106 Fab bound to DIII revealed only nine contact residues, from the A-strand (K307, K310), the end of the B-strand (K325, Y326), and the connecting BC (E327, T329, D330) and DE (K361, E362) loops (**Fig. 1A** **and** **Table 1**); these results are consistent with prior mapping data by yeast surface display, which implicated five of these residues as essential recognition determinants (K310, T329, D330, K361 and E362) [13]. Yeast surface display results also implicated G328, P332, and P364 in E106 binding, and mutation of any of these residues would likely result in an altered presentation of the direct contact residues. Charge reversals at either E384 or K385 in the FG-loop also diminished E106 recognition, and this loop is adjacent to the primary E106 epitope. Overall, the contact residues contributed 24 van der Waal interactions, 14 hydrogen bonds, and 10 electrostatic interactions to the interface (**Table S1**). The E106 structural footprint represents a unique composite of previously identified DIII-specific neutralizing epitopes on flaviviruses including the lateral ridge (N-terminal region, BC, DE and FG loops) [19], [20] and A-strand epitopes [10], [21]. DENV-1 DIII was engaged by 11 heavy chain residues from CDR1 (I30, G31, Y32 and Y33), CDR2 (N52, E50, and R53), and CDR3 (R95, I196, N97 and W98) (**Fig. 1B** **, top panel**) and four light chain residues from CDR1 (D30, D32), CDR2 (E50), and CDR3 (L94) (**Table S1**).

**Table 1:** Data collection and refinement statistics^a^.

Wavelength	0.976 Å
Resolution	20 – 2.45 Å (2.54-2.45 Å)
Number of observed reflections	166 322
Unique reflections	26 014
Redundancy	6.4 (4.8)
I/σI	19.8 (2.1)
*R*merge^b^	0.083 (0.546)
Completeness	99.5% (95.9%)
Spacegroup	P2_1_2_1_2_1_
Cell dimensions	a = 82.7 Å, b = 91.8 Å, c = 92.6 Å; α = β = γ = 90°
*Refinement statistics*	
Resolution	20-2.45 Å (2.55 - 2.45 Å)
No. of reflections/No. in *R_free_*	25912/1134
R*_cryst_*/R*_free_* c	18.9%/23.9%
No. atoms protein/solvent	4063/94
<B> protein	59.2 Å^2^
<B> solvent	49.3 Å^2^
Rmsd bond lengths	0.004 Å
Rmsd bond angles	0.822°
Ramachandran favoured	97.5%
Ramachandran allowed	2.5%
Ramachandran outliers	0%
Molprobity score^d^	1.07
Molprobity clash score	1.99
PDB ID	4L5F

a Numbers in parentheses refer to the highest resolution shell.

b R_merge_ = Σ|I−<I>|/Σ<I>, where I is the intensity of each individual reflection.

c *R* = ∑(F_P_−F_calc_)/∑F_P_.

d Molprobity score defined as 0.42574 * log(1+clashscore)+0.32996 * log(1+max(0,pctRotOut-1))+0.24979 * log(1+max(0,100−pctRamaFavored-2))+0.5.

A comparison of the DIII structure in complex with Fab versus unbound DIII revealed small conformational differences, with a root mean square displacement of 0.9 Å in the α-carbons over 98 residues. Of the DIII residues that directly interacted with the E106 Fab, the greatest differences in α-carbon positions involved residue T329 (1.2 Å); this was significant because a recently identified E106 MAb neutralization escape mutant showed a T to A amino acid change at position 329 (**Fig. 1B** **, bottom panel**) and [22]).

The E106 structural epitope on DIII was characterized by a high shape complementarity score (*Sc* = 0.73, with a perfect fit being 1) [23], which is greater than typical antibody-antigen interactions (*Sc* = 0.64–0.68) [23] but similar to anti-flavivirus MAb-E protein interactions (**Table 2**). The combined surface area buried by the DIII-E106 Fab complex was ∼1,243 Å^2^ (**Fig. 1C** and **Table 2**) [24] which is less than most antibody-antigen (1,400–2,300 Å^2^) [25], [26] and anti-flavivirus MAb-E protein interactions (**Table 2**). Typical of many antibody-antigen complexes, the majority (∼70%) of the DIII-E106 Fab interface was contributed by the heavy chain (**Fig. 1C**), with a combined buried surface of 877 Å^2^ (401 Å^2^ of the heavy chain and 476 Å^2^ of domain III). The light chain contributed the remaining buried surface (172 Å^2^ of the light chain and 194 Å^2^ of domain III).

**Table 2:** DENV-1 DIII-E106 Fab structural interface.

Ab*^a^* – Ag*^b^* complex	Ag (Å^2^)	Ab (Å^2^)	Ag+Ab (Å^2^)	Sc*^c^*
E106 – DENV1 DIII	670	573	1 243	0.73
E16 – WNV DIII	789	810	1 599	0.76
1A1D-2 – DENV2 DIII	914	936	1 850	0.44
E53 – WNV E	596	581	1 177	0.66
E111 – DENV1 DIII	1010	1085	2095	0.68
2H12 – DENV1 DIII	652	630.5	1305.2	0.76
2H12 – DENV3 DIII	589	544	1132	0.79
2H12 – DENV4 DIII	518–548	464–488	982–1036	0.76–0.79
4E11 – DENV1 DIII	877	910	1787	0.71
4E11 – DENV2 DIII	886	876	1762	0.73
4E11 – DENV3 DIII	723–775	742–751	1474–1517	0.77
4E11 – DENV4 DIII	883	879	1762	0.74

The structural interface of DENV-1 DIII-E106 Fab (described by the surface area of antibody, Ab*^a^* and antigen, Ag*^b^* that is buried [24] as well as shape complementarity, Sc*^c^* in the complex [23]) is compared to previously determined anti-DENV and anti-WNV Fab complexes. PDB codes E16 – WNV E DIII, 1ZTX [20]; 1A1D-2 – DENV2 E DIII, 2R29 [21]; E53 – WNV E, 3I50 [43]; E111 – DENV1 E DIII, 4FFY [33]; 2H12 – DENV1 DIII, 4AL8 [17]; 2H12 – DENV3 DIII, 4ALA [17]; 2H12 – DENV4 DIII, 4AM0 [17]; 4E11 – DENV1 DIII, 3UZQ [10]; 4E11 – DENV2 DIII, 3UZV [10]; 4E11 – DENV3 DIII, 3UZE [10]; 4E11 – DENV4 DIII, 3UYP [10].

All nine DIII contact residues were conserved in the five DENV-1 genotypes (K361 is replaced by the conservative substitution R361 in genotype 5 strains); this likely explains why E106 neutralized infection of all five DENV-1 genotypes efficiently (**Fig. 1D and 1E**), and [13]). In comparison, only one of the nine contact residues (Y326) was conserved in DENV-2, DENV-3, DENV-4 or WNV, a finding that is consistent with virological data showing that DENV-1-E106 MAb neutralizes infection in a serotype-specific manner [13]. E106 binds to DENV-4 but not to DENV-2 or DENV-3 [13]. The number of conserved contact residues did not correlate with DENV serotype binding specificity (DENV-4 has four whereas DENV-2 and DENV-3 each have five, **Fig. 1E**), which may instead be accounted for by other factors, including differential maturation [**27**] or relative virion dynamics [28].

### E106 Fab binds DIII with micromolar monovalent affinity

**Figure 2:**
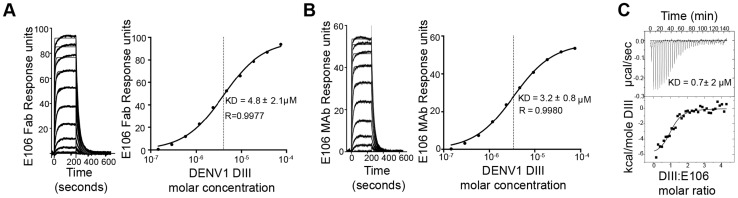
E106 Fab and MAb binds with low affinity to DENV-1-DIII. Analysis of DENV-1 DIII binding to (**A**) E106 Fab and (**B**) E106 MAb as measured by SPR. The kinetically fit sensorgrams (fits in gray, raw data in black) for which a 5.8±3.0 µM affinity (Fab) and 4.2±1.2 µM (MAb) was calculated on the left panel is similar to the equilibrium data fit 4.8±2.1 µM (Fab) and 3.2±0.8 µM (MAb) which is shown on the right panel. (**C**) ITC confirms a micromolar affinity of E106 Fab for DENV-1 DIII. Results are representative of at least two independent experiments.

To determine the significance of the small buried interface of our E106-DIII complex, we investigated E106 binding to DIII by surface plasmon resonance (SPR). Increasing concentrations of purified DENV-1 DIII monomer were flowed over immobilized E106 Fab (**Fig. 2A**). Equilibrium analysis surprisingly revealed a micromolar affinity (4.8±2.1 µM) for this interaction. A similar result was observed when increasing concentrations of DENV-1-DIII monomer was flowed over immobilized intact E106 MAb in the solid phase (3.2±0.8 µM, **Fig. 2B**); this experiment eliminates the possibility that papain cleavage and the removal of the E106 Fc region altered the monovalent binding parameters. Binding to the ectodomain of DENV-1 E (DI-DII-DIII) also appeared equivalently weak (1.1±0.1 µM). As an independent measurement of affinity, we performed isothermal titration calorimetry under similar experimental conditions as SPR by injecting DENV-1 DIII into a solution of E106 Fab. Using this method with completely unmodified proteins we again measured micromolar affinity for the E106 Fab/DIII interaction (K_D_ = 0.7±2 µM) (**Fig. 2C**).

### E106 Fab binds and neutralizes DENV-1 poorly

**Figure 3:**
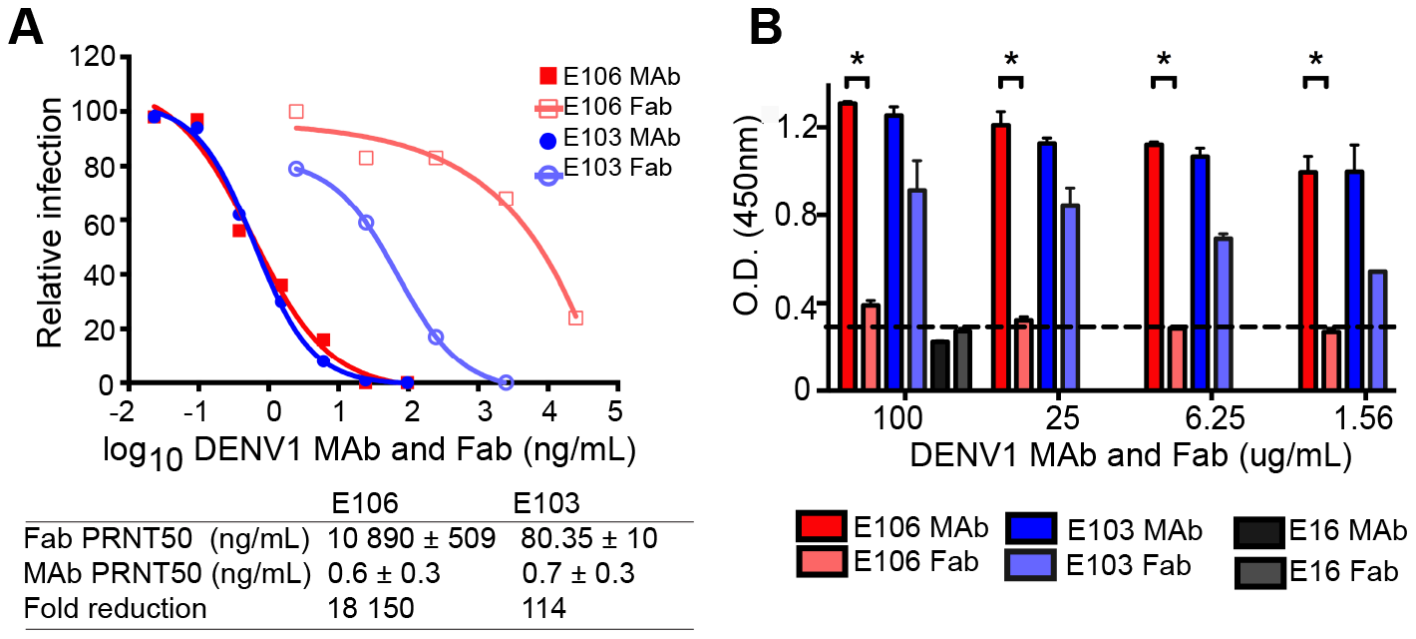
Fab versus MAb neutralization and binding. (**A**) DENV-1 neutralization by E106 MAb (filled square, red) and Fab (empty square, salmon) (upper panel) and corresponding fold reduction (lower panel). The control E103 MAb (filled circle, blue) and corresponding Fab (empty circle, purple) is included for comparison. (**B**) Qualitative ELISA binding of MAbs and Fabs to DENV-1. E106 MAb binds virions to a similar extent as E103 MAb but E106 Fab binding is significantly less than E103 Fab (P<0.0001). The negative control is E16. The results are from two ELISA experiments performed in duplicate and without background subtraction. The limit of detection was determined by performing the assay in the absence of virus.

The micromolar monovalent affinity of the highly therapeutic E106 antibody was unanticipated in light of its picomolar inhibitory activity (4±2 pM or 0.6±0.3 ng/mL; our most potent neutralizing anti-flavivirus MAb isolated to date); as a comparison, our therapeutic DIII-specific anti-WNV MAb E16 (inhibitory activity 30 to 80 pM or 4–18 ng/mL), which has advanced to human clinical trials [29], has a monovalent affinity of 3.4 nM [30]. We hypothesized that while E106 MAb potently inhibited DENV-1 infection, Fabs should lack this activity. To test this, we compared the ability of Fab and intact IgG from E106 and E103, a lateral ridge DIII-specific DENV-1 neutralizing antibody [13], to inhibit infection. While monovalent E103 Fab showed a 114-fold decrease in neutralization potency compared to the intact bivalent IgG, the Fab of E106 Fab showed a remarkable 18,150-fold decrease in inhibitory activity when compared to intact IgG (**Fig. 3A**).

To investigate this observation further, we performed a virion-binding assay by ELISA. DENV-1 virions (strain 16007) were captured with humanized DIII A-strand-specific antibody (DENV-1-E50) [31] and then detected with Fab or intact IgG of E103, E106, or WNV E16 (negative control). Notably, the amount of virus detected with E106 IgG was indistinguishable from E103 IgG (**Fig. 3B**). In comparison, E106 Fab bound virions to a significantly lower level (*P*<0.0001) at all concentrations tested, than those derived from E103 (**Fig. 3B**). Thus, disparate neutralization of E106 MAb and Fab correlated with discordant binding to DENV-1 virions and was consistent with the biophysical measurements: monovalent binding by DENV-1-E106 is surprisingly inefficient given the potent inhibitory activity of intact antibody.

### E106 MAb binds bivalently to DENV-1 virus particles

**Figure 4:**
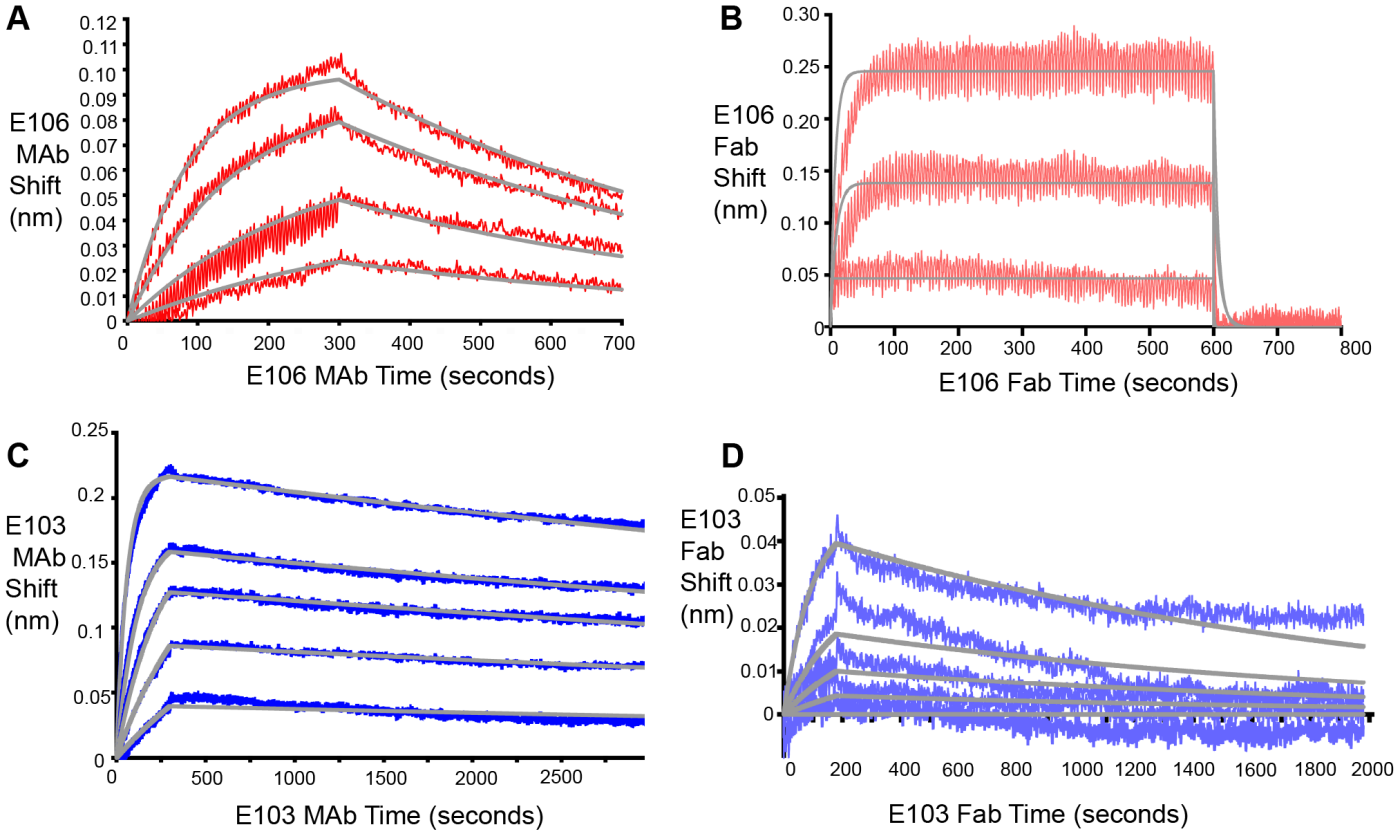
Fab and MAb binding to DENV-1 RVPs. (**A**) E106 MAb binding to DENV-1 RVPs by bio-layer interferometry. E106 MAb binds with high apparent affinity to DENV-1 RVPs (K_Dapp_ of 13±2 nM) with a slow dissociation (t_1/2_>400 sec). Raw data is in red and fits are in gray. (**B**) Direct binding of E106 Fab to DENV-1 RVPs. Results are representative of several independent experiments that showed low affinity binding (K_D_ of >1 µM) and fast off-rate kinetics (t_1/2_<2 sec). Raw data is in salmon and fits are in gray. BLI determined binding of the control E103 (**C**) MAb and (**D**) Fab to DENV-1 RVPs. E103 MAb and Fab binding to DENV-1 RVPs are comparable, with a calculated apparent K_Dapp_ of 0.8±0.1 nM and K_D_ of 7±1 nM, respectively, as expected for a predominantly monovalent interaction. Raw data is in blue (E103 MAb) and purple (E103 Fab) and fits are in gray. Results are representative of at least two independent experiments.

Based on these experiments, we hypothesized that efficient neutralization of DENV-1 infection by E106 required bivalent binding. Using bio-layer interferometry (BLI), we measured the affinity and kinetics of MAb and Fab binding to intact DENV-1 virus particles [32] (**Fig. 4**). E106 MAb (**Fig. 4A**) bound DENV-1 particles with an apparent affinity (avidity), K_Dapp_ of 13±2 nM. In contrast, E106 Fab had an affinity of K_D_>1 µM, with a rapid dissociation rate (t_1/2_<2 sec) that was at least 800-fold faster than E106 MAb (t_1/2_>400 sec) (**Fig. 4B**). These results contrast with more comparable binding of E103 MAb (K_Dapp_ of 0.8±0.1 nM) (**Fig. 4C**) and E103 Fab (K_D_ of 7±1 nM) to DENV-1 particles (**Fig. 4D**). Importantly, the binding affinities of E106 Fab engaging isolated DIII measured by SPR and ITC is remarkably similar to that observed by BLI for the binding to DENV-1 virions, suggesting that our structurally defined DIII epitope corresponds to the entire virion surface recognized by a single Fab.

### Mechanistic correlates of E106 MAb neutralization

**Figure 5:**
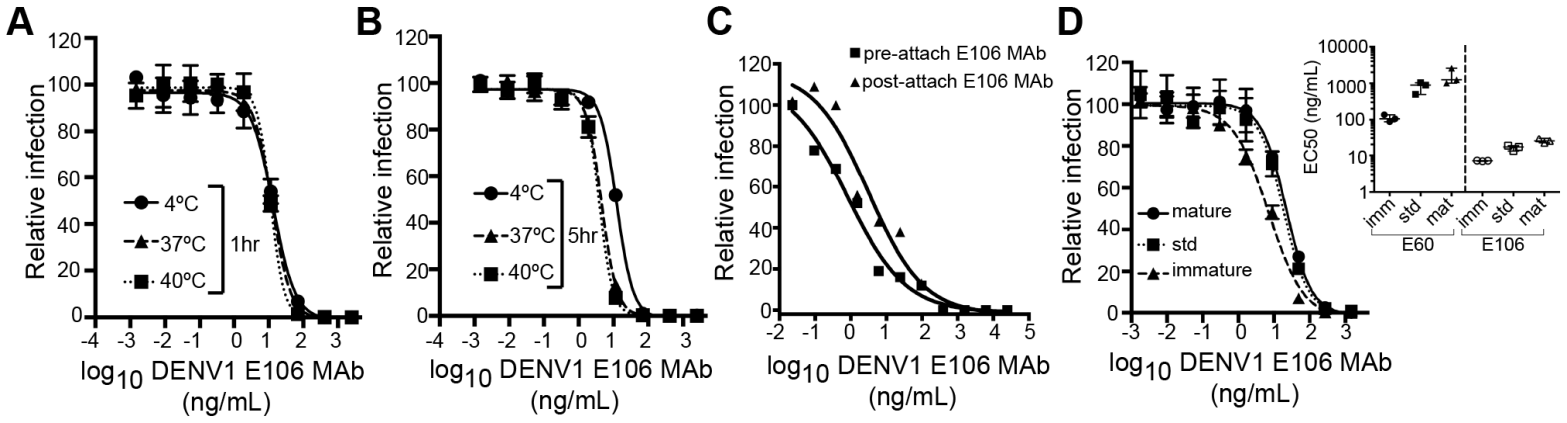
Functional characteristics of neutralization by E106 MAb. (**A–B**) Time and temperature dependence of neutralization. DENV-1 RVPs were pre-incubated with E106 MAb for one (**A**) or five hours (**B**) at three different temperatures (4°C, 37°C and 40°C) before infecting Raji-DCSIGNR cells. Infection was carried out at 37°C and assessed 48 h later by flow cytometry. Error bars represent the standard error of the mean of duplicate infections. Results are representative of two independent experiments. (**C**) Pre- or post-attachment neutralization test. BHK21-15 cells were pre-chilled to 4°C, and 10^2^ PFU of DENV-1 (strain 16007) was added to each well for one hour at 4°C. After extensive washing at 4°C, increasing concentrations of E106 MAb were added for one hour at 4°C, and the PRNT determined (triangles, Post). A standard pre-incubation PRNT with all steps performed at 4°C is shown for reference (squares, Pre). Data shown are representative from three experiments performed in duplicate. (**D**) E106 MAb neutralization is insensitive to the maturation state of the virus. E106 MAb neutralization of immature, standard, and mature 16007 RVP preparations, independently validated with the fusion-loop specific E60 MAb, are shown. A representative neutralization assay of three experiments is depicted. Error bars represent the standard error of the mean of duplicate infections. The inset depicts the EC50 values of neutralization of the distinct RVP preparations (immature, standard and mature) by E106 MAb and the control E60 MAb.

We next investigated the time- and temperature-dependence of E106 neutralization, as this analysis can provide information as to the relative accessibility of epitopes [28], [33]. Changes in the time or temperature of incubation did not appreciably affect E106 neutralization (**Fig. 5A and B**). By performing pre- and post-attachment neutralization assays, we found that, similar to several other potently neutralizing DIII-specific antibodies against flaviviruses [15], [30], [34], E106 can neutralize infection even after virus attaches to cells (**Fig. 5C**). Finally, we tested the neutralization of E106 MAb as a function of the maturation state of the virus. DENV virions are a heterogeneous mixture of immature, partially mature and fully mature virions, with immature virions being generally less or non-infectious. In this assay, E106 MAb neutralization proved to be independent of the maturation state of the virus (**Fig. 5D**). In comparison, neutralization by E60, a DII fusion-loop-specific MAb, was sensitive to virion maturation, as seen previously [**27**].

### Modeling of E106 binding to virus

**Figure 6:**
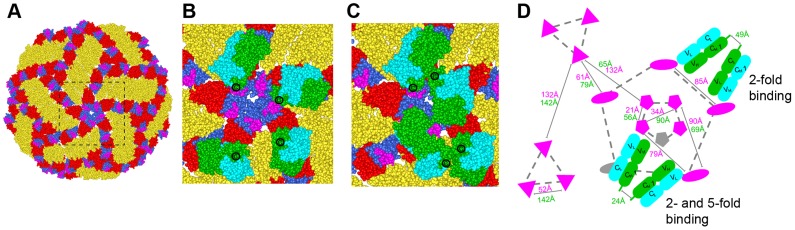
Modeling of E106 binding to DENV. (**A**) The E106 epitope is highlighted in magenta on a DENV-1 model of the DENV-2 cryo-electron microscopic reconstruction (PDB code 1P58). All atoms of the model are displayed and colored by domains (DI, red; DII, gold; DIII, blue). Magnified view of the boxed region in (A) with E106 Fab docked onto the epitopes at the primary candidates for bivalent antibody binding namely (**B**) adjacent 2-fold epitopes (49 Å CH1 separation) which is permuted as an outer 5-fold ring and (**C**) adjacent inner and outer 5-fold epitopes (24 Å CH1 separation). E106 Fab heavy and light chain is represented by green and cyan spheres, respectively. The C-terminal CH1 residue is represented by ‘C’. (**D**) Schematic of the possible arrangements of E106 MAb bivalent binding to the lateral ridge plus A-strand epitope (magenta) on a single virion. The distances labeled in magenta and green are E106 epitope (T329) and C-terminal CH1 residue (R214) distances respectively. One Fab pair (green, heavy chain; cyan, light chain) is shown across the 5- and 2-fold and another involves adjacent 2-fold symmetry axes (the 2-fold permutes as an outer 5-fold ring, dashed gray line). 5-, 3- and 2-fold axes of symmetry are connected by gray dashed lines for clarity and represented by pentagons, triangles, and ovals, respectively. Epitopes that are occluded as a result of bivalent binding are shown in gray.

In contrast to non-enveloped viruses where a structural understanding of bivalent antibody binding has emerged [35]–[37], there currently is no such data for icosahedral enveloped viruses including flaviviruses. To address how E106 might recognize DENV-1 bivalently we docked our structure onto the cryo-EM derived model of the mature DENV virion (**Fig. 6**) [38]. While the E106 epitope is predominantly exposed on all 180 E protein monomers (**Fig. 6A**), unimpeded binding is readily apparent only on monomers in the 3-fold and 2-fold symmetry axes, similar to what we observed for E16 binding to WNV [20], [39]. However, minor reorientation of DIII subunits on the inner 5-fold symmetry axis would allow for up to two E106 Fabs to bind there at the same time as three Fabs could bind to the outer 5-fold (2-fold) related epitopes. We measured the distance separating the docked Fab CH1 domain C-termini with the expectation that distances greater than 50 Å would be unlikely spanned by 16 hinge residues [36], [37]. This analysis indicated the possibility for limited bivalent bridging, with the primary candidates being adjacent outer 5-fold epitopes (49 Å CH1 separation) (**Fig. 6B and D**) as well as adjacent inner and outer 5-fold epitopes (24 Å CH1 separation) (**Fig. 6C and D**). These epitopes are 85 Å and 79 Å apart, respectively, within the expected reach of a single IgG molecule (117–134±40 Å) [40]. We also examined the E106 epitope on the recent cryo-EM reconstruction of DENV-2 at 37°C [41], [42]; this ‘bumpy’ virion supports a similar model of bivalent binding to DIII on the 5-fold and 2-fold symmetry axes.

## Discussion

Epitope mapping studies have enhanced our understanding of the mechanisms of virus neutralization and identified sites on the E protein of flaviviruses that are targeted by neutralizing antibodies [7]. These include the lateral ridge of DIII of WNV and JEV [19], [20], the A-strand of DIII of DENV [10], [21], the CC′ loop of DIII of DENV-1 [33], the fusion loop of DII of WNV and DENV [43], a DI epitope of DENV-4 [11], and a complex epitope centered at the hinge of DI and DII on WNV [44] and DENV [45], [46]. Here, we describe a composite epitope, comprised of regions of the lateral ridge and A-strand of DIII that is targeted by the therapeutic MAb E106. DIII residues contacted by E106 were highly conserved among DENV-1 genotypes but variable in other DENV serotypes. Consistent with this, E106 potently neutralized all five DENV-1 genotypes, but not other DENV serotypes nor WNV [13].

The E106 Fab-DIII complex was characterized by a small-buried interface, which correlated with an unexpectedly weak micromolar affinity, as determined both by SPR and isothermal calorimetry. We found no evidence for E106 binding to residues outside of DIII as the binding affinity to recombinant DIII appears to be very similar to the binding of Fab to E ectodomain protein or virions; consistent with this, neutralization escape studies only identified residues in DIII [22]. Monovalent E106 Fab poorly neutralized DENV-1 compared to intact E106 IgG, and this finding correlated with poor binding of Fab to intact virus. Although our structural models suggest that E106 can readily bind the isolated pre-fusion dimer and post-fusion trimer and possibly prevent the ∼70° transition of DIII that is associated with membrane fusion [47], [48], the inability of E106 Fab fragments to neutralize virus efficiently argues against this model. Our data are more consistent with a mechanism of neutralization that requires bivalent binding of E106 IgG to single virions, and cross-linking of E protein monomers in adjacent symmetry groups to prevent requisite E protein rearrangements during infection.

The measurement of micromolar monovalent affinity was unexpected given that E106 is our most potently neutralizing and protective anti-DENV-1 MAb (EC50 of 0.6 ng/ml against DENV-1 strain 16007), which is at least 10-fold more potent than our well-characterized DIII-lateral ridge-specific therapeutic MAb (E16) against WNV [20], [30]. Indeed, E106 had the lowest EC50 value of ∼500 anti-flavivirus MAbs (DENV-1, DENV-2, DENV-3, DENV-4, and WNV) generated to date in our laboratory [13], [15], [30], [49]. Is there a correlation between neutralization potency and E106 bivalent binding to single virions? The icosahedral arrangement of the E protein on the mature DENV virion displays 180 copies of the E protein. Our *in silico* modeling predicts that in one possible arrangement, up to 48 of these sites may be available for bivalent engagement by 24 intact E106 IgG. Since monovalent binding was insufficient for neutralization, bivalent binding to single virions could neutralize infection by inhibiting an essential stage of the virus lifecycle (attachment, entry, or fusion). Alternatively, bivalent binding across virions could neutralize DENV infection by aggregating virus. Our post-attachment studies suggest that E106 MAb was capable of neutralizing infection even after virus attached to the cell surface. Aggregation also appears less likely because the neutralization curves did not show a characteristic triphasic dose-response curve that was reported in inhibition studies describing antibody-virus aggregation [50]. We favor a model in which bivalent binding of E106 stabilizes and/or cross-links one or more E protein monomers in different symmetry groups, analogous to monovalent binding of WNV CR4354 MAb [44], and thus prevents radial expansion and rearrangement that is requisite for fusion of viral and host endosomal membranes [48], [51].

E106 is one of very few MAbs that have been shown to require bivalent binding for efficient virus neutralization [35], [36], [52], and the first one directed against a flavivirus. While antibodies can be multivalent, with the potential to bind to virus particles with high avidity, the relatively small number of bivalent binding MAbs described to date may be attributed to the following: (i) the limited number of epitopes displayed on a single particle for some viruses; (ii) the position and orientation of epitopes that are beyond the reach of a bivalent MAb, which is limited by torsional flexibility of its hinge; (iii) the radius of curvature of virions, which may restrict the accessibility of epitopes by MAb (iv) post-translational modification (e.g. N-linked glycans) of virions that may hinder bivalent MAb engagement; (v) immunization protocols that rely on isolated recombinant envelope proteins, rather than envelope proteins in the structurally unique form of the intact virion; and (vi) assays that do not screen for bivalent neutralizing MAbs. E106 was generated after priming and boosting with infectious DENV-1 [13]. The repetitive E antigens in the icosahedral orientation of the virion may have facilitated selection of low monovalent affinity yet high avidity antibodies. While some antibodies against HIV have been described as bivalent, they actually are bispecific, with each arm binding distinct epitopes [53]. This is likely due to the paucity of trimeric spikes on the surface of the virus (∼14) and their irregular spacing [54]. Although several MAbs have been proposed to require bivalent binding for efficient virus neutralization [55], [56], compelling evidence is presented only for the non-enveloped positive strand RNA viruses, specifically, human rhinovirus 2 [36] and 14 [35], [52] and the rabbit hemorrhagic disease calicivirus [37]. It may be that the quasi-icosahedral arrangement of the flavivirus envelope creates a landscape that permits limited bivalent MAb engagement.

Bivalent engagement of the virion by antibodies could be an important concept for DENV vaccine development. Immunity against DENV may not be achieved optimally using a subunit vaccine approach, as isolated E protein monomers may not induce antibodies that require bivalent binding for strong binding and neutralization. Analogously, some human MAbs against WNV bind a complex epitope at two independent positions on adjacent E protein monomers in different symmetry groups, which is only present on an intact WNV particle [44]. Human MAbs isolated from DENV-infected individuals are believed to recognize similar quaternary epitopes in E that are present only in the context of the intact DENV virion [45], [46]. Given that E106 MAb was our most potent and therapeutic anti-DENV-1 MAb, strategies that enhance the likelihood of generating and identifying neutralizing antibodies that function through bivalent binding mechanisms may improve the potency of inhibitory humoral responses against DENV and other flaviviruses. Regardless, an understanding of the structural and mechanistic basis for the neutralization activity of E106 MAb provides new insights into the humoral response against flaviviruses.

## Materials and Methods

### E106 Fab-DIII crystal structure determination

DENV-1 DIII (residues 293 to 399 of the E protein of strain 16007) was expressed in bacteria and re-folded oxidatively from isolated inclusion bodies as described previously [13]. Fab fragments of E106 were prepared using immobilized papain resin according to the manufacturer's instructions (Pierce). MAb (5 to 10 mg) was digested for 18 hours at 37°C, and passed over a protein A agarose resin to remove Fc fragments and undigested MAb and purified on a S-75 size exclusion chromatography column equilibrated in 20 mM HEPES pH 7.4 and 150 mM NaCl. Antibody–antigen complexes were formed by mixing papain-generated, gel filtered E106 Fab fragments with DIII at a ratio of 1.2∶1 and crystallized by the hanging drop vapor diffusion method at a total protein concentration of 14 mg/mL in a solution of 22% PEG 6,000, and 0.1 M MES pH 5.0 (final pH 5.7). Crystals (in 1 µL crystallization drops) were cryoprotected by the addition of 0.2 µL aliquots of cryobuffer (in 23.5% PEG 6,000, 0.1 M MES pH 5.0, final pH 5.7, and 20% glycerol), then transferred to a fresh drop of cryobuffer and rapidly cooled in liquid nitrogen. X-ray diffraction data were collected at ALS beamline 4.2.2 (Lawrence Berkeley Laboratories) at a wavelength of 0.976 Å at 100 K with a CCD detector, and indexed and scaled in HKL2000 [57]. The crystals diffracted to 2.45 Å resolution and belonged to the space group *P*2_1_2_1_2_1_ with unit cell dimensions of a = 82.7 Å, b = 91.8 Å, c = 92.6 Å, with one E106 Fab-DIII complex per asymmetric unit. Crystallographic phasing was obtained by molecular replacement using the program Phaser [58] and the coordinates of DENV-1 DIII (Protein Data Bank (PDB) 3EGP) and the Fab fragment of CTM01 IgG (PDB 1AD9). Iterative model building and refinement was performed using Coot [59] and Refmac [60] and later Phenix [61]. The final structure was refined to R_cryst_ = 18.9% and R_free_ = 23.9%. The final model includes DENV-1 DIII amino acid residues 297 to 394, E106 heavy chain residues 1 to 214 (Chothia numbering), and light chain residues 1 to 213. The atomic coordinates and structure factors of E106 Fab bound to DENV-1 DIII (CSGID target number IDP00272) have been deposited in the Protein Data Bank (www.rcsb.org) under PDB accession number 4L5F. Structural figures were prepared using CCP4MG [62] and Pymol [63] (surface representation using 1.4 Å solvent probe) and where shown, spheres represent van der Waal radii, vdw * 1.1.

### Surface plasmon resonance and isothermal titration calorimetry

Monovalent antibody affinity analysis was performed using SPR (BIAcore T100, GE Company) and ITC (VP-ITC instrument, Microcal) at 10°C in 50 mM HEPES, pH 7.5 and 100 mM NaCl. For SPR, E106 MAb or Fab was immobilized at low concentrations (∼500 Response Units) to a CM5 chip (GE healthcare) using amine-coupling chemistry. Bacterially-expressed DIII (residues 293–399) of DENV-1 (strain 16007) was injected at a flow rate of 65 µl/min at concentrations ranging from 0.2 µM to 500 µM for three minutes to saturate binding and then allowed to dissociate for seven minutes. The half-life of the monovalent interaction was short and did not require additional regeneration of the chip surface in preparation for the next DIII injection. The observed binding curves were double referenced to a non-reactive antibody (WNV E16) as well as buffer in the absence of DIII. Curves were analyzed by a steady-state fit for a 1∶1 interaction, and a nonlinear least squares fit was used to evaluate the fit of the curve to the observed data. Alternatively, 500 response units (RU) of DIII were immobilized onto a CM5 chip and E106 Fab fragments were injected to saturation. Affinity measurements of E106 for the DENV-1 E ectodomain (DI-DII-DIII) were performed such that insect-derived DENV-1 E glycoprotein (ProSpec-Tany TechnoGene Ltd.) was in the stationary phase and E106 Fab fragments were in the mobile phase, in order to conserve limited protein and avoid avidity affects. Additional regeneration was not necessary because of the short half-life for the interaction. WNV E ectodomain was used as a negative control for E106 binding. Analysis was performed as with the DIII described above. ITC experiments were performed such that 4 to 8 µL of DENV-1-DIII protein (90 to 110 µM) was injected into 1.4 mL of E106 Fab protein (6 to 7 µM) over a total of 36 injections. The titration data were integrated and normalized in Origin (Microcal) to determine the reaction stoichiometry, *n*, and equilibrium constant *Ka* ( = *K* ^−1^ *_d_*).

### Neutralization assays and capture ELISA

Plaque reduction neutralization tests (PRNT) and pre- and post-attachment neutralization assays were performed with DENV-1 strain 16007 on Vero cells as previously described [13], [64]. Binding of intact MAbs or Fabs (E103, E106, and a negative control WNV E16) to DENV-1 virions (strain 16007) was detected by capture ELISA [13], [64]. Briefly, humanized DENV-1 E50 MAb (subcomplex DIII A-strand specific antibody) was coated at 2 µg/ml on MaxiSorp (Nunc) polystyrene 96-well microtiter plates in a sodium carbonate (pH 9.3) buffer. Plates were washed three times in wash buffer (PBS with 0.02% Tween 20) and blocked for one hour at 37°C with blocking buffer (DMEM with 10% FBS). DENV-1 virions (2.5×10^5^ PFU) diluted in DMEM with 10% heat-inactivated FBS were captured on plates for two hours at 37°C. Wells were washed thrice with blocking buffer and DENV-1 MAb or Fab was then added at 100 µg/ml and 4-fold serial dilutions to duplicate wells and incubated for two hours at 37°C. Plates were washed five times and then sequentially incubated with goat anti-mouse (whole molecule) IgG-HRP (Sigma, St Louis, MO) and tetramethylbenzidine substrate (Dako). The reaction was stopped with the addition of 2 N H_2_SO_4_ to the medium, and emission (450 nm) was read using an iMark microplate reader (Bio-Rad).

### Time and temperature-dependent neutralization of DENV-1

A plasmid expressing the C-prM-E genes of DENV-1 (strain 16007) was co-transfected into HEK-293T cells with a plasmid encoding a WNV replicon expressing GFP. Transfected cells were incubated at 30°C and RVP harvested at 72 and 96 hours post-transfection, filtered through a 0.2 µM filter, and stored aliquoted at −80°C. DENV-1 RVP were incubated with serial dilutions of MAb under conditions of antibody excess at 4°C, 37°C, or 40°C for one or five hours. Subsequently, MAb-RVP mixtures were added to Raji-DCSIGNR cells and incubated at 37°C for 48 hours. Infected cells were assayed for GFP expression using a BD FACSCalibur flow cytometer as described [28].

### The kinetics of DENV-1 RVP binding to DENV-1 MAbs and Fabs

All bio-layer interferometry studies were performed in PBS buffer supplemented with 1 to 2 mg/ml BSA (PBS-B) at 25°C using an Octet Red biosensor system (ForteBio). DENV-1 reporter virus particles (RVPs) (Western Pacific 74 strain) were produced as previously described [65]. To purify virus particles, RVP production supernatant was harvested, clarified through a 0.22 µm filter (Corning), and PEG precipitated using 7.5% PEG 8000 (Sigma). The virus particles were further purified through two 20% sucrose cushions before resuspension in HBS. Samples were stored at −80°C and gently thawed prior to use. RVPs were loaded onto streptavidin (SA) biosensor tips using a human monoclonal antibody against DENV-1 (1H7.2, the anti-prM antibody, a gift from James Crowe), which was captured using a biotinylated goat anti-human polyclonal antibody (GAH Fc, Southern Biotech). Briefly, GAH Fc was diluted to 5 µg/ml in PBS-B and bound to SA sensor tips for five minutes. Following a brief rinse in PBS-B, 1H7.2 (5 µg/ml in PBS-B) was captured for five minutes. After another brief rinse, DENV-1 RVPs diluted to 10 µg/ml (or 50 µg/ml (E106 Fab)) were loaded for 45 minutes followed by a five-minute stabilization. Antibody association was measured for up to 10 minutes followed by dissociation for 20 minutes (E106) or 45 minutes (E103) in buffer. Non-specific binding was assessed using sensor tips without RVPs as well as using sensor tips loaded with retroviral pseudotypes (Lipoparticles) containing only endogenous cell surface receptors (no viral envelope protein). Data analysis was performed using Octet Data Analysis v6.4 (ForteBio). Binding kinetics were analyzed using a standard 1∶1 binding model.

## Supporting Information

Table S1 **E106/DENV1-DIII interface.**
(DOCX)

## References

[1] Bhatt S, Gething PW, Brady OJ, Messina JP, Farlow AW, et al (2013) The global distribution and burden of dengue. Nature 496: 504–507 doi:10.1038/nature12060 23563266 10.1038/nature12060 PMC3651993

[2] Rico-Hesse R (1990) Molecular evolution and distribution of dengue viruses type 1 and 2 in nature. Virology 174: 479–493 doi:10.1016/0042-6822(90)90102-W 2129562 10.1016/0042-6822(90)90102-w

[3] Holmes EC, Twiddy SS (2003) The origin, emergence and evolutionary genetics of dengue virus. Infect Genet Evol 3: 19–28 doi:S1567134803000042 [pii] 12797969 10.1016/s1567-1348(03)00004-2

[4] Sabin AB (1952) Research on dengue during World War II. Am J Trop Med Hyg 1: 30–50. 14903434 10.4269/ajtmh.1952.1.30

[5] Reich NG, Shrestha S, King AA, Rohani P, Lessler J, et al (2013) Interactions between serotypes of dengue highlight epidemiological impact of cross-immunity. J R Soc Interface 10 doi:10.1098/rsif.2013.0414 10.1098/rsif.2013.0414 PMC3730691 23825116

[6] Halstead SB (1989) Antibody, macrophages, dengue virus infection, shock, and hemorrhage: a pathogenetic cascade. Rev Infect Dis 11 Suppl 4: S830–9 doi:10.1111/j.1742-4658.2005.04870.x 2665015 10.1093/clinids/11.supplement_4.s830

[7] Pierson TC, Fremont DH, Kuhn RJ, Diamond MS (2008) Structural insights into the mechanisms of antibody-mediated neutralization of flavivirus infection: implications for vaccine development. Cell Host Microbe 4: 229–238 doi:S1931-3128(08)00260-6 [pii] 10.1016/j.chom.2008.08.004 18779049 10.1016/j.chom.2008.08.004 PMC2678546

[8] Rey FA, Heinz FX, Mandl C, Kunz C, Harrison SC (1995) The envelope glycoprotein from tick-borne encephalitis virus at 2 A resolution. Nature 375: 291–298 doi:10.1038/375291a0 7753193 10.1038/375291a0

[9] Modis Y, Ogata S, Clements D, Harrison SC (2003) A ligand-binding pocket in the dengue virus envelope glycoprotein. Proc Natl Acad Sci U S A 100: 6986–6991 doi:10.1073/pnas.0832193100 0832193100 [pii] 12759475 10.1073/pnas.0832193100 PMC165817

[10] Cockburn JJ, Navarro Sanchez ME, Fretes N, Urvoas A, Staropoli I, et al (2012) Mechanism of dengue virus broad cross-neutralization by a monoclonal antibody. Structure 20: 303–314 doi:S0969-2126(12)00005-6 [pii] 10.1016/j.str.2012.01.001 22285214 10.1016/j.str.2012.01.001

[11] Cockburn JJ, Navarro Sanchez ME, Goncalvez AP, Zaitseva E, Stura EA, et al (2011) Structural insights into the neutralization mechanism of a higher primate antibody against dengue virus. EMBO J 31: 767–779 doi:emboj2011439 [pii] 10.1038/emboj.2011.439 22139356 10.1038/emboj.2011.439 PMC3273384

[12] Gromowski GD, Barrett AD (2007) Characterization of an antigenic site that contains a dominant, type-specific neutralization determinant on the envelope protein domain III (ED3) of dengue 2 virus. Virology 366: 349–360 doi:S0042-6822(07)00379-0 [pii] 10.1016/j.virol.2007.05.042 17719070 10.1016/j.virol.2007.05.042

[13] Shrestha B, Brien JD, Sukupolvi-Petty S, Austin SK, Edeling MA, et al (2010) The development of therapeutic antibodies that neutralize homologous and heterologous genotypes of dengue virus type 1. PLoS Pathog 6: e1000823 doi:10.1371/journal.ppat.1000823 20369024 10.1371/journal.ppat.1000823 PMC2848552

[14] Wahala WM, Donaldson EF, De Alwis R, Accavitti-Loper MA, Baric RS, et al (2010) Natural strain variation and antibody neutralization of dengue serotype 3 viruses. PLoS Pathog 6: e1000821 doi:10.1371/journal.ppat.1000821 20333252 10.1371/journal.ppat.1000821 PMC2841629

[15] Sukupolvi-Petty S, Austin SK, Engle M, Brien JD, Dowd KA, et al (2010) Structure and function analysis of therapeutic monoclonal antibodies against dengue virus type 2. J Virol 84: 9227–9239 doi:JVI.01087-10 [pii] 10.1128/JVI.01087-10 20592088 10.1128/JVI.01087-10 PMC2937608

[16] Roehrig JT, Bolin RA, Kelly RG (1998) Monoclonal antibody mapping of the envelope glycoprotein of the dengue 2 virus, Jamaica. Virology 246: 317–328 doi:S0042-6822(98)99200-5 [pii] 10.1006/viro.1998.9200 9657950 10.1006/viro.1998.9200

[17] Midgley CM, Flanagan A, Tran HB, Dejnirattisai W, Chawansuntati K, et al (2012) Structural analysis of a dengue cross-reactive antibody complexed with envelope domain III reveals the molecular basis of cross-reactivity. J Immunol 188: 4971–4979 doi:10.4049/jimmunol.1200227 22491255 10.4049/jimmunol.1200227 PMC3364712

[18] Thompson BS, Moesker B, Smit JM, Wilschut J, Diamond MS, et al (2009) A therapeutic antibody against west nile virus neutralizes infection by blocking fusion within endosomes. PLoS Pathog 5: e1000453 doi:10.1371/journal.ppat.1000453 19478866 10.1371/journal.ppat.1000453 PMC2679195

[19] Wu KP, Wu CW, Tsao YP, Kuo TW, Lou YC, et al (2003) Structural basis of a flavivirus recognized by its neutralizing antibody: solution structure of the domain III of the Japanese encephalitis virus envelope protein. J Biol Chem 278: 46007–46013 doi:10.1074/jbc.M307776200 M307776200 [pii] 12952958 10.1074/jbc.M307776200

[20] Nybakken GE, Oliphant T, Johnson S, Burke S, Diamond MS, et al (2005) Structural basis of West Nile virus neutralization by a therapeutic antibody. Nature 437: 764–769 doi:nature03956 [pii] 10.1038/nature03956 16193056 10.1038/nature03956 PMC7095628

[21] Lok SM, Kostyuchenko V, Nybakken GE, Holdaway HA, Battisti AJ, et al (2008) Binding of a neutralizing antibody to dengue virus alters the arrangement of surface glycoproteins. Nat Struct Mol Biol 15: 312–317 doi:nsmb.1382 [pii] 10.1038/nsmb.1382 18264114 10.1038/nsmb.1382

[22] Shrestha B, Austin SK, Dowd KA, Prasad AN, Youn S, et al (2012) Complex phenotypes in mosquitoes and mice associated with neutralization escape of a Dengue virus type 1 monoclonal antibody. Virology 427: 127–134 doi:S0042-6822(12)00105-5 [pii] 10.1016/j.virol.2012.02.010 22406169 10.1016/j.virol.2012.02.010 PMC3312934

[23] Lawrence MC, Colman PM (1993) Shape complementarity at protein/protein interfaces. J Mol Biol 234: 946–950 doi:S0022-2836(83)71648-7 [pii] 10.1006/jmbi.1993.1648 8263940 10.1006/jmbi.1993.1648

[24] Krissinel E, Henrick K (2007) Inference of macromolecular assemblies from crystalline state. J Mol Biol 372: 774–797 doi:S0022-2836(07)00642-0 [pii] 10.1016/j.jmb.2007.05.022 17681537 10.1016/j.jmb.2007.05.022

[25] Davies DR, Padlan EA, Sheriff S (1990) Antibody-antigen complexes. Annu Rev Biochem 59: 439–473 doi:10.1146/annurev.bi.59.070190.002255 2197980 10.1146/annurev.bi.59.070190.002255

[26] Sundberg EJ, Mariuzza RA (2002) Molecular recognition in antibody-antigen complexes. Adv Protein Chem 61: 119–160. 12461823 10.1016/s0065-3233(02)61004-6

[27] Nelson S, Jost CA, Xu Q, Ess J, Martin JE, et al (2008) Maturation of West Nile Virus Modulates Sensitivity to Antibody-Mediated Neutralization. PLoS Pathog 4: e1000060 doi:10.1371/journal.ppat.1000060 18464894 10.1371/journal.ppat.1000060 PMC2330159

[28] Dowd KA, Jost CA, Durbin AP, Whitehead SS, Pierson TC (2011) A dynamic landscape for antibody binding modulates antibody-mediated neutralization of West Nile virus. PLoS Pathog 7: e1002111 doi:10.1371/journal.ppat.1002111 21738473 10.1371/journal.ppat.1002111 PMC3128118

[29] Beigel JH, Nordstrom JL, Pillemer SR, Roncal C, Goldwater DR, et al (2010) Safety and Pharmacokinetics of Single Intravenous Dose of MGAWN1, a Novel Monoclonal Antibody to West Nile Virus. Antimicrob Agents Chemother 54: 2431–2436 doi:10.1128/AAC.01178-09 20350945 10.1128/AAC.01178-09 PMC2876374

[30] Oliphant T, Engle M, Nybakken GE, Doane C, Johnson S, et al (2005) Development of a humanized monoclonal antibody with therapeutic potential against West Nile virus. Nat Med 11: 522–530 doi:nm1240 [pii] 10.1038/nm1240 15852016 10.1038/nm1240 PMC1458527

[31] Rodrigo WWSI, Block OKT, Lane C, Sukupolvi-Petty S, Goncalvez AP, et al (2009) Dengue virus neutralization is modulated by IgG antibody subclass and Fcgamma receptor subtype. Virology 394: 175–182 doi:10.1016/j.virol.2009.09.024 19833371 10.1016/j.virol.2009.09.024 PMC2783259

[32] Ansarah-Sobrinho C, Nelson S, Jost CA, Whitehead SS, Pierson TC (2008) Temperature-dependent production of pseudoinfectious dengue reporter virus particles by complementation. Virology 381: 67–74 doi:S0042-6822(08)00525-4 [pii] 10.1016/j.virol.2008.08.021 18801552 10.1016/j.virol.2008.08.021 PMC3428711

[33] Austin SK, Dowd KA, Shrestha B, Nelson CA, Edeling MA, et al (2012) Structural Basis of Differential Neutralization of DENV-1 Genotypes by an Antibody that Recognizes a Cryptic Epitope. PLoS Pathog 8: e1002930 doi:10.1371/journal.ppat.1002930 23055922 10.1371/journal.ppat.1002930 PMC3464233

[34] Stiasny K, Kiermayr S, Holzmann H, Heinz FX (2006) Cryptic properties of a cluster of dominant flavivirus cross-reactive antigenic sites. J Virol 80: 9557–9568 doi:10.1128/JVI.00080-06 16973559 10.1128/JVI.00080-06 PMC1617264

[35] Smith TJ, Olson NH, Cheng RH, Chase ES, Baker TS (1993) Structure of a human rhinovirus-bivalently bound antibody complex: implications for viral neutralization and antibody flexibility. Proc Natl Acad Sci USA 90: 7015–7018 doi:10.1073/pnas.90.15.7015 8394005 10.1073/pnas.90.15.7015 PMC47066

[36] Hewat EA, Blaas D (1996) Structure of a neutralizing antibody bound bivalently to human rhinovirus 2. EMBO J 15: 1515–1523. 8612574 PMC450059

[37] Thouvenin E, Laurent S, Madelaine M-F, Rasschaert D, Vautherot J-F, et al (1997) Bivalent binding of a neutralising antibody to a calicivirus involves the torsional flexibility of the antibody hinge. Journal of Molecular Biology 270: 238–246 doi:10.1006/jmbi.1997.1095 9236125 10.1006/jmbi.1997.1095

[38] Zhang W, Chipman PR, Corver J, Johnson PR, Zhang Y, et al (2003) Visualization of membrane protein domains by cryo-electron microscopy of dengue virus. Nat Struct Mol Biol 10: 907–912 doi:10.1038/nsb990 10.1038/nsb990 PMC4148076 14528291

[39] Kaufmann B, Nybakken GE, Chipman PR, Zhang W, Diamond MS, et al (2006) West Nile virus in complex with the Fab fragment of a neutralizing monoclonal antibody. Proc Natl Acad Sci U S A 103: 12400–12404 doi:0603488103 [pii] 10.1073/pnas.0603488103 16895988 10.1073/pnas.0603488103 PMC1567891

[40] Sosnick TR, Benjamin DC, Novotny J, Seeger PA, Trewhella J (1992) Distances between the antigen-binding sites of three murine antibody subclasses measured using neutron and X-ray scattering. Biochemistry 31: 1779–1786 doi:10.1021/bi00121a028 1737031 10.1021/bi00121a028

[41] Fibriansah G, Ng T-S, Kostyuchenko VA, Lee J, Lee S, et al (2013) Structural changes in dengue virus when exposed to a temperature of 37°C. J Virol 87: 7585–7592 doi:10.1128/JVI.00757-13 23637405 10.1128/JVI.00757-13 PMC3700303

[42] Zhang X, Sheng J, Plevka P, Kuhn RJ, Diamond MS, et al (2013) Dengue structure differs at the temperatures of its human and mosquito hosts. Proc Natl Acad Sci USA 110: 6795–6799 doi:10.1073/pnas.1304300110 23569243 10.1073/pnas.1304300110 PMC3637732

[43] Cherrier MV, Kaufmann B, Nybakken GE, Lok SM, Warren JT, et al (2009) Structural basis for the preferential recognition of immature flaviviruses by a fusion-loop antibody. EMBO J 28: 3269–3276 doi:emboj2009245 [pii] 10.1038/emboj.2009.245 19713934 10.1038/emboj.2009.245 PMC2771083

[44] Kaufmann B, Vogt MR, Goudsmit J, Holdaway HA, Aksyuk AA, et al (2010) Neutralization of West Nile virus by cross-linking of its surface proteins with Fab fragments of the human monoclonal antibody CR4354. Proc Natl Acad Sci U S A 107: 18950–18955 doi:1011036107 [pii] 10.1073/pnas.1011036107 20956322 10.1073/pnas.1011036107 PMC2973864

[45] De Alwis R, Smith SA, Olivarez NP, Messer WB, Huynh JP, et al (2012) Identification of human neutralizing antibodies that bind to complex epitopes on dengue virions. Proc Natl Acad Sci USA 109: 7439–7444 doi:10.1073/pnas.1200566109 22499787 10.1073/pnas.1200566109 PMC3358852

[46] Teoh EP, Kukkaro P, Teo EW, Lim APC, Tan TT, et al (2012) The structural basis for serotype-specific neutralization of dengue virus by a human antibody. Sci Transl Med 4: 139ra83 doi:10.1126/scitranslmed.3003888 10.1126/scitranslmed.3003888 22723463

[47] Kuhn RJ, Zhang W, Rossmann MG, Pletnev SV, Corver J, et al (2002) Structure of dengue virus: implications for flavivirus organization, maturation, and fusion. Cell 108: 717–725 doi:S0092867402006608 [pii] 11893341 10.1016/s0092-8674(02)00660-8 PMC4152842

[48] Modis Y, Ogata S, Clements D, Harrison SC (2004) Structure of the dengue virus envelope protein after membrane fusion. Nature 427: 313–319 doi:10.1038/nature02165 nature02165 [pii] 14737159 10.1038/nature02165

[49] Brien JD, Austin SK, Sukupolvi-Petty S, O'Brien KM, Johnson S, et al (2010) Genotype-specific neutralization and protection by antibodies against dengue virus type 3. J Virol 84: 10630–10643 doi:JVI.01190-10 [pii] 10.1128/JVI.01190-10 20702644 10.1128/JVI.01190-10 PMC2950583

[50] Thomas AA, Vrijsen R, Boeye A (1986) Relationship between poliovirus neutralization and aggregation. J Virol 59: 479–485. 3016307 10.1128/jvi.59.2.479-485.1986 PMC253099

[51] Kaufmann B, Chipman PR, Holdaway HA, Johnson S, Fremont DH, et al (2009) Capturing a flavivirus pre-fusion intermediate. PLoS Pathog 5: e1000672 doi:10.1371/journal.ppat.1000672 19956725 10.1371/journal.ppat.1000672 PMC2776511

[52] Smith TJ, Olson NH, Cheng RH, Liu H, Chase ES, et al (1993) Structure of human rhinovirus complexed with Fab fragments from a neutralizing antibody. J Virol 67: 1148–1158. 7679742 10.1128/jvi.67.3.1148-1158.1993 PMC237479

[53] Mouquet H, Scheid JF, Zoller MJ, Krogsgaard M, Ott RG, et al (2010) Polyreactivity increases the apparent affinity of anti-HIV antibodies by heteroligation. Nature 467: 591–595 doi:nature09385 [pii] 10.1038/nature09385 20882016 10.1038/nature09385 PMC3699875

[54] Zhu P, Liu J, Bess J, Chertova E, Lifson JD, et al (2006) Distribution and three-dimensional structure of AIDS virus envelope spikes. Nature 441: 847–852 doi:nature04817 [pii] 10.1038/nature04817 16728975 10.1038/nature04817

[55] Schofield DJ, Stephenson JR, Dimmock NJ (1997) Variations in the neutralizing and haemagglutination-inhibiting activities of five influenza A virus-specific IgGs and their antibody fragments. J Gen Virol 78 Pt 10: 2431–2439. 9349461 10.1099/0022-1317-78-10-2431

[56] Klasse PJ, Sattentau QJ (2002) Occupancy and mechanism in antibody-mediated neutralization of animal viruses. J Gen Virol 83: 2091–2108. 12185262 10.1099/0022-1317-83-9-2091

[57] Otwinowski Z, Minor W (1997) Processing of X-ray diffraction data collected in oscillation mode. In: C.WCarter J& RMS, editors. Methods in Enzymology. New York: Academic Press. pp. 307–326. 10.1016/S0076-6879(97)76066-X 27754618

[58] McCoy AJ, Grosse-Kunstleve RW, Adams PD, Winn MD, Storoni LC, et al (2007) Phaser crystallographic software. J Appl Crystallogr 40: 658–674 doi:10.1107/S0021889807021206 19461840 10.1107/S0021889807021206 PMC2483472

[59] Emsley P, Cowtan K (2004) Coot: model-building tools for molecular graphics. Acta Crystallogr D Biol Crystallogr 60: 2126–2132 doi:S0907444904019158 [pii] 10.1107/S0907444904019158 15572765 10.1107/S0907444904019158

[60] Murshudov GN, Vagin AA, Dodson EJ (1997) Refinement of macromolecular structures by the maximum-likelihood method. Acta Crystallogr D Biol Crystallogr 53: 240–255 doi:10.1107/S0907444996012255 S0907444996012255 [pii] 15299926 10.1107/S0907444996012255

[61] Adams PD, Afonine PV, Bunkóczi G, Chen VB, Davis IW, et al (2010) PHENIX: a comprehensive Python-based system for macromolecular structure solution. Acta Crystallographica Section D Biological Crystallography 66: 213–221 doi:10.1107/S0907444909052925 20124702 10.1107/S0907444909052925 PMC2815670

[62] Potterton L, McNicholas S, Krissinel E, Gruber J, Cowtan K, et al (2004) Developments in the CCP4 molecular-graphics project. Acta Crystallogr D Biol Crystallogr 60: 2288–2294 doi:10.1107/S0907444904023716 15572783 10.1107/S0907444904023716

[63] DeLano WL (2008) The PyMOL Molecular Graphics System, version 1. Schrödinger, LLC (Oregon).

[64] Vogt MR, Moesker B, Goudsmit J, Jongeneelen M, Austin SK, et al (2009) Human monoclonal antibodies against West Nile virus induced by natural infection neutralize at a postattachment step. J Virol 83: 6494–6507 doi:JVI.00286-09 [pii] 10.1128/JVI.00286-09 19386704 10.1128/JVI.00286-09 PMC2698525

[65] Mattia K, Puffer BA, Williams KL, Gonzalez R, Murray M, et al (2011) Dengue reporter virus particles for measuring neutralizing antibodies against each of the four dengue serotypes. PLoS ONE 6: e27252 doi:10.1371/journal.pone.0027252 22096543 10.1371/journal.pone.0027252 PMC3212561

