# Supplementary material for: Potent Dengue Virus Neutralization by a Therapeutic Antibody with Low Monovalent Affinity Requires Bivalent Engagement
Source: PLoS Pathog. 2014 Apr 17;10(4):e1004072. doi: 10.1371/journal.ppat.1004072 (PMC3990716; doi:10.1371/journal.ppat.1004072)
Supplement: Table S1 — E106/DENV1-DIII interface. (DOCX) [file ppat.1004072.s001.docx]

**Table S1. E106/DENV1-DIII interface**

| **DIII** | | | **E106** | **Distance(Å)** |
| --- | --- | --- | --- | --- |
| *Van der Waals contacts^a^* | | | | |
| Lys^E307^ (Cβ) | Trp^H98^ (CH2) | | | 3.59 |
| Lys^E307^ (Cβ) | Trp^H98^ (Cζ3) | | | 3.90 |
| Lys^E325^  (Cε) | Trp^H98^ (Cε2) | | | 3.59 |
| Lys^E325^ (Cε) | Trp^H98^ (Cδ1) | | | 3.70 |
| Lys^E325^ (Cε) | Trp^H98^ (Cδ2) | | | 3.86 |
| Tyr^E326^ (C) | Trp^H98^ (Cζ2) | | | 3.76 |
| Glu^E327^ (Cβ) | Trp^H98^ (CH2) | | | 3.89 |
| Glu^E327^ (Cβ) | Trp^H98^ (Cζ2) | | | 3.65 |
| Glu^E327^ (Cβ) | Trp^H98^ (Cε2) | | | 3.88 |
| Glu^E327^ (Cδ) | Ile^H96^ (Cα) | | | 3.73 |
| Thr^E329^ (Cβ) | Asn^H52^ (Cγ) | | | 3.68 |
| Thr^E329^ (Cγ2) | Tyr^H33^ (Cε1) | | | 3.75 |
| Thr^E329^ (Cγ2) | Tyr^H33^ (Cδ1) | | | 3.65 |
| Thr^E329^ (Cγ2) | Tyr^H32^ (C) | | | 3.71 |
| Thr^E329^ (Cγ2) | Tyr^H32^ (Cα) | | | 3.76 |
| Thr^E329^ (Cγ2) | Gly^H31^ (C) | | | 3.59 |
| Asp^E330^ (Cβ) | Arg^H53^ (Cζ) | | | 3.88 |
| Lys^E361^ (Cε) | Tyr^H33^  (Cζ) | | | 3.32 |
| Lys^E361^ (Cε) | Tyr^H33^ (Cε2) | | | 3.74 |
| Lys^E361^ (Cδ) | Tyr^H33^ (Cζ) | | | 3.67 |
| Lys^E361^ (Cε) | Tyr^H33^ (Cε1) | | | 3.68 |
| Lys^E361^ (Cδ) | Tyr^H33^ (Cε1) | | | 3.45 |
| Glu^E362^ (Cδ) | Leu^L94^ (Cδ1) | | | 3.76 |
| Glu^E362^ (Cδ) | Leu^L94^ (Cγ) | | | 3.77 |
| *Electrostatic interactions^b^* | | | | |
| Lys^E310^ (Nζ) | Asp^L30^ (Oδ2) | | | 2.75 |
| Lys^E310^ (Nζ) | Asp^L30^ (Oδ1) | | | 3.02 |
| Lys^E325^ (Nζ) | Asp^L32^ (Oδ1) | | | 3.51 |
| Lys^E325^ (Nζ) | Asp^L32^ (Oδ2) | | | 3.88 |
| Lys^E325^ (Nζ) | Glu^L50^ (Oε1) | | | 2.69 |
| Glu^E362^ (Oε2) | Arg^H95^ (NH1) | | | 3.88 |
| Lys^E361^ (Nζ) | Glu^H50^ (Oε2) | | | 3.03 |
| Lys^E361^ (Nζ) | Glu^H50^ (Oε1) | | | 3.51 |
| Lys^E325^ (Nζ) | Glu^L50^ (Oε2) | | | 3.20 |
| Lys^E307^ (Nζ) | Glu^L50^ (Oε1) | | | 3.50 |
| *Direct hydrogen bonds^c^* | | | | |
| Lys^E325^ (Nζ) | | Glu^L50^ (Oε1) | | 2.69 |
| Glu^E327^ (Oε1) | | Asn^H97^ (N) | | 2.87 |
| Glu^E327^ (Oε2) | | Trp^H98^  (N) | | 2.68 |
| Thr^E329^ (O) | | Asn^H52^ (Nδ2) | | 2.97 |
| Thr^E329^ (Oγ1) | | Ile^H30^  (O) | | 2.66 |
| Thr^E329^ (Oγ1) | | Arg^H53^ (NH2) | | 2.81 |
| Thr^E329^ (Oγ1) | | Arg^H53^ (Nε | | 2.97 |
| Lys^E310^(Nζ) | | Asp^L30^ (Oδ2) | | 2.75 |
| Lys^E361^ (Nζ | | Glu^H50^ (Oε2) | | 3.03 |
| *Indirect (solvent mediated) hydrogen bonds^c^* | | | | |
| Glu^E362^ (Oε1) | | Leu^L94^ (N) | |  |
| Lys^E361^ (O) | | Trp^H98^ (Nε1) | |  |
| Glu^E362^ (Oε1) | | Arg^H95^ (NH1) | |  |
| Glu^E362^ (Oε2) | | Arg^H95^ (NH2) | |  |
| Glu^E327^ (Oε2) | | Arg^H95^ (Nε) | |  |

*^a^*Van der Waals interactions defined in Ligplot [1], *^b^*electrostatic interactions as defined by PISA [2] and *^c^*hydrogen bonds as defined by HBPLUS [3] in Ligplot.

**REFERENCES**

1. Wallace AC, Laskowski RA, Thornton JM (1995) LIGPLOT: a program to generate schematic diagrams of protein-ligand interactions. Protein Eng 8: 127–134.

2. Krissinel E, Henrick K (2007) Inference of macromolecular assemblies from crystalline state. J Mol Biol 372: 774–797. doi:S0022-2836(07)00642-0 [pii] 10.1016/j.jmb.2007.05.022.

3. McDonald IK, Thornton JM (1994) Satisfying hydrogen bonding potential in proteins. J Mol Biol 238: 777–793. doi:S0022-2836(84)71334-9 [pii] 10.1006/jmbi.1994.1334.
